# Supplementary material for: Levels of pneumococcal conjugate vaccine coverage and indirect protection against invasive pneumococcal disease and pneumonia hospitalisations in Australia: An observational study
Source: PLoS Med. 2021 Aug 3;18(8):e1003733. doi: 10.1371/journal.pmed.1003733 (PMC8376256; doi:10.1371/journal.pmed.1003733)
Supplement: S1 Text — (DOCX) [file pmed.1003733.s002.docx]

Determining the pneumococcal vaccination coverage required for indirect effects against invasive pneumococcal disease and pneumonia hospitalisations in Australia

DATA ANALYSIS PLAN

| Study Investigators | Jocelyn Chan, Cattram Nguyen, Heather Gidding, Ross Andrews, Sanjay Jayasinghe, Peter McIntyre, Fiona Russell, Chris Blyth, Hannah Moore, Parveen Fathima, Kim Mulholland |
| --- | --- |
| Version date | 2 April 2018 |

# Background

The introduction of pneumococcal vaccines in Australia have seen overall rates of invasive pneumococcal disease (IPD) in all age groups decline by nearly 50% from 2002 to 2014. While the indirect effects of PCV are well described, including in Australia, the vaccination coverage required to achieve indirect benefits is unknown.

## Summary of PCV introduction in Australia [1]

The 7-valent PCV (PCV7), given at 2, 4, and 6 months (3+0 schedule) was introduced into Australia’s National Immunisation Program from July 2001 for Aboriginal and Torres Strait Islander and at-risk children, alongside a 12-18 month booster with the polysaccharide pneumococcal vaccine. From January 2005, PCV7 was introduced for all infants using the same 3+0 schedule. In July 2011, the 13-valent PCV (PCV13) replace PCV7 at 2,4 and 6 months of age, this was a accompanied by a catch-up program in which children 12-35 months of age who had completed a primary PCV7 course received a supplementary dose of PCV13. In October 2012, a booster dose of PCV13 was introduced for Indigenous children at 12-18 months of age in NT, QLD, SA and WA.

# Objectives

Our objectives are:

1. To investigate the relationship between community-level PCV7 coverage and PCV7-type IPD among under-vaccinated children
2. To investigate the relationship between community-level PCV13 coverage and PCV13-type, non-PCV7-type IPD among under-vaccinated children
3. To investigate the relationship between community-level PCV7/PCV13 coverage and pneumonia hospitalization among under-vaccinated children

# Study cohort

The full study cohort consists of 1.95 million children from two states, New South Wales and Western Australia, born between 1996 and 2012, with linked health and vaccination data until December 2013. National death and immunisation databases, along with state-based health data (notifications, perinatal data,) were probabilistically linked using demographic details. Due the lack of a dynamic population register, immigrants were not included in this study cohort.

For this analysis we will only be using children born between 1 January 2003 and 31 December 2012 (see table 1). See Appendix A for more details on construction of dataset.

Table 1: Datasets to be used in the analysis

| **Dataset name and location** | **Birth cohort** | **WA** | **NSW** | **Total** |
| --- | --- | --- | --- | --- |
| Analysis > Pneumonia hospitalisations > pneumo_cohort_base_final | 1 Jan 2001 - 31 Dec 2012* | 326 406 | 1 039 490 | 1 365 896 |

*Noting that hospitalisations in NSW only available from July 2001

# Study variables and endpoints

## Outcome variables

The primary outcome variables for the analysis are:

1. Invasive pneumococcal disease notification
   1. PCV7-type IPD* - serotypes 4, 6B, 9V, 14, 18C, 19F, and 23F
   2. PCV13, non-PCV7-type IPD - serotypes 1, 3, 5, 6A, 7F, and 19A

*IPD is defined as is defined as isolation of Streptococcus pneumoniae by culture or detection of nucleic acid from a normally sterile. Date of IPD is defined as earliest onset date. Obtained through disease notification data.

1. Pneumonia hospitalisation
   1. presumptive pneumococcal pneumonia hospitalisation
   2. all-cause pneumonia hospitalisation
   3. pneumonia hospitalisation due to unspecified cause
   4. pneumonia hospitalisation due to other specified cause

Records will be classified into the above categories based on the hospitalisation codes detailed in Appendix B – coding is according to the 10th Revision of the International Classification of Diseases Australian Modification (ICD-10-AM).

## Exposure variables

The primary exposure variable is population-level vaccination coverage which will be a time-varying covariate. Each child’s person-time will be split by quarter of calendar time (1 Jan – 31 Mar, 1 Apr – 30 Jun, 1 Jul – 30 Sep, 1 Oct -31 Dec), and for each quarter, we will determine the PCV coverage among children 12-23 months of age in the child’s statistical local area (SLA) of residence at the commencement of that time period (table 2).

Table 2: Definition of pneumococcal conjugate vaccine coverage

| **Vaccine** | **Definition of PCV coverage** |
| --- | --- |
| PCV7 | Proportion of children under five years of age at the commencement of each quarter who have received at least two doses of any PCV at any age or at least one dose at greater than 12 months of age |
| PCV13 | Proportion of children under five years of age at the commencement of each quarter who have received at least two doses of PCV13 at any age or at least one dose at greater than 12 months of age |

To determine the degree to which our definition for PCV coverage affects relationship between vaccine coverage and IPD, we will perform a sensitivity analysis using receipt of three doses of PCV as the definition for comparison. Alternative methods for defining PCV coverage are listed in Appendix C.

## Covariates

The following covariates are available within the dataset. Further discussion of model selection will be included in the analysis section below.

Table 3: List of available covariates

| **Variable** | **Data type** | **Data source/Comments** |
| --- | --- | --- |
| Gender | Binary, stable | Male/Female |
| Gestational age | Binary, stable | Term (>=28 weeks)/ preterm (<28 weeks) |
| Low birth weight | Binary, stable | Yes (<2.5kg) / No (>=2.5kg) |
| SEIFA Index of Relative Disadvantage (SLA level) | Categorical, stable | 0-10%/11-25%/26-75%/76-89%/90-100%. Based on state specific cut offs. |
| ARIA (Accessibility/Remoteness Index of Australia, SLA level) | Categorical, stable | Very remote, Remote, Outer regional, Inner regional, Metropolitan |
| Indigenous status | Binary, stable | Indigenous/non-Indigenous child – defined using an algorithm by Christensen et al., which assigns Indigenous status if two or more linked records are coded as Indigenous |
| State of birth | Binary, stable | WA/NSW |
| Statistical Local Area (SLA) of mother’s residence at birth | Categorical, stable | Text |
| Postcode of mother’s residence at birth | Categorical, stable | 4-digit code |
| Local Government Area (LGA) of mother’s residence at birth | Categorical, stable | Text |
| Season at each quarter | Categorical, time-varying | Winter (Apr-Sep) / Summer (Oct-Mar) |
| Any hospitalisation for Cat A* or Cat B** condition before IPD onset | Binary, stable | Yes/No |
| Season of birth | Categorical, stable | Summer, Autumn, Winter, Spring |
| Mum age at baby birth | Continuous, stable |  |
| Father age at baby birth | Continuous, stable |  |
| Mother’s country of birth | Categorical, stable |  |
| Parity | Categorical stable | **Number of previous pregnancies** |
| Apgar score | Categorical, stable | **Apgar score at 5 minutes** |
| Delivery method | Categorical, stable | **1=Vaginal, 2=Instrumental, 3=Caesarean, 9=missing** |
| Smoking during pregnancy | Binary, stable | **1=Smoked during pregnancy, 0=No smoking during pregnancy** |

*Cat A condition – defined according to the immunisation handbook as functional or anatomical asplenia, immunocompromising conditions, proven or presumptive cerebrospinal fluid (CSF) leak, cochlear implants, intracranial shunts

**Cat B condition – defined according to the immunisation handbook as chronic cardiac disease, chronic lung disease, diabetes mellitus, Down syndrome, chronic liver disease

# Proposed analytical strategy

## Descriptive analyses

### Description of IPD data

We will graph rates of PCV7-type and PCV13, non-PCV7-type IPD per 100 000 year person-time among Indigenous and non-Indigenous under-vaccinated children 0-59 months of age in NSW and WA over each quarter from the 1 January 2002 to 31 December 2013.

We define a child as under-vaccinated if the child has received less than the adequate number of doses to have had a protective immune response against vaccine serotypes. We define an adequate number of immunisations for a child <12 months, as receiving two or more PCV doses. For children 12 months of age or older, a child is vaccinated if they received two PCV doses in the first year of life or at least one PCV dose after the age of 12 months, otherwise they are under-vaccinated.

### Description of pneumonia hospitalisation data

We will graph rates of pneumonia hospitalisation per 100 000 year person time among Indigenous and non-Indigenous under-vaccinated children 0-59 months of age in NSW and WA at the commencement of each quarter from 1 January 2002 to 31 December 2013

### Description of vaccine data

We will graph coverage of any PCV (i.e. PCV7 or PCV13) among children 12-23 months of age in NSW and WA at the commencement of each quarter from the 1 January 2002 to 31 December 2013.

We will also graph coverage of PCV13 among children 12-23 months of age in NSW and WA at the commencement of each quarter from the 1 January 2002 to 31 December 2013.

## Primary analysis

### Setting the data

For the purposes of this analysis only under-vaccinated children will contribute person-time. We will use time since birth (i.e. age) as the primary time axis for all analyses since risk of IPD or pneumonia hospitalisation is strongly correlated with age.

To be classified as vaccinated against serotypes included in PCV7, the child must have received an adequate number of any PCV doses. To be classified as vaccinated against serotypes included in PCV13, the child must have received an adequate number of PCV13-specific doses.

The start and end date for person time are listed in the table below. Each child’s person time will be split quarterly to allow for season and SLA-level PCV coverage to vary over time.

SLAs with fewer than 50 children aged five years of age younger will be excluded from the analysis.

Table 4: Birth cohort, study period and censoring points for PCV7 and PCV13 analyses

| **Analysis** | **Birth cohort** | **Study period, & start person-time (if not birth)** | **Primary outcome** | **Analysis specific censoring points, & end person time** |
| --- | --- | --- | --- | --- |
| IPD, PCV7 period | 1 Jan 2003-31 Dec 2008 | 1 Jan 2003-31 Dec 2008 | PCV7-type IPD | End person time at:  - age 59 mths (or 5yrs) on receipt of two doses of any PCV at any age or receipt of one dose at greater than 12 months of age  - study end date  - death  - outcome |
| IPD PCV13 period | 1 Jan 2010-31 Dec 2013 | 1 Jan 2010-31 Dec 2013 | PCV13, non-PCV7-type IPD | End person time at:  - age 59 mths (or 5yrs) on receipt of two doses of PCV13 at any age or receipt of one dose at greater than 12 months of age  - study end date  - death  - outcome |
| Pneumonia hospitalisation, PCV7 period | 1 Jan 2003-31 Dec 2008 | 1 Jan 2003-31 Dec 2008 | No. of hospitalizations for:  - presumptive pneumococcal pneumonia  - all-cause pneumonia  - pneumonia due to specified cause  - pneumonia due to unspecified cause | End person time at:  - age 59 mths (or 5yrs) on receipt of two doses of any PCV at any age or receipt of one dose at greater than 12 months of age  - study end date  - death  - outcome |
| Pneumonia hospitalisation, PCV13 period | 1 Jan 2010-31 Dec 2013 | 1 Jan 2010-31 Dec 2013 |  | End person time at:  - age 59 mths (or 5yrs) on receipt of two doses of PCV13 at any age or receipt of one dose at greater than 12 months of age  - study end date  - death  - outcome |

### Poisson regression

We will construct the following individual level poisson regression models to investigate the relationship between quarterly PCV coverage at child’s SLA of residence (exposure) and the rate of the following primary outcomes:

1. PCV7-type IPD
2. PCV13, non-PCV7-type IPD
3. Presumptive pneumococcal pneumonia hospitalisation
4. All-cause pneumonia hospitalisation
5. Pneumonia hospitalisation due to a specified cause
6. Pneumonia hospitalisation due to an unspecified cause

The rate ratio reported would describe the relative rate decrease for each increase in percentage point of SLA-level vaccination coverage. We have chosen Poisson regression analyses of ungrouped person-time data to allow for reporting of baseline rates and rate ratios, as well as allowing for adjustment by individual level risk factors. [2]

To identify confounders for adjustment, we constructed directed acyclic graphs (DAG). DAGs include all variables potentially related to exposure and outcome, connected using uni-directional arrows showing causal relationships between variables. The graph identifies potentially confounding pathways and allows investigators to determine variables that should be controlled for to obtain unbiased effect estimates. Potential confounders were identified from relevant literature. [3, 4]

Based on the DAG below, we have identified age, season at birth, season, time since PCV introduction, ARIA index (rurality), Indigenous status and the SEIFA score (socioeconomic status) as the minimally sufficient confounding subset for adjustment (figure 1).

Note that censoring on vaccine receipt may introduce bias due to informative censoring since children are more likely to be vaccinated earlier over time (and when SLA-level vaccination coverage is higher) – however this is accounted for by adjustment factors listed above.


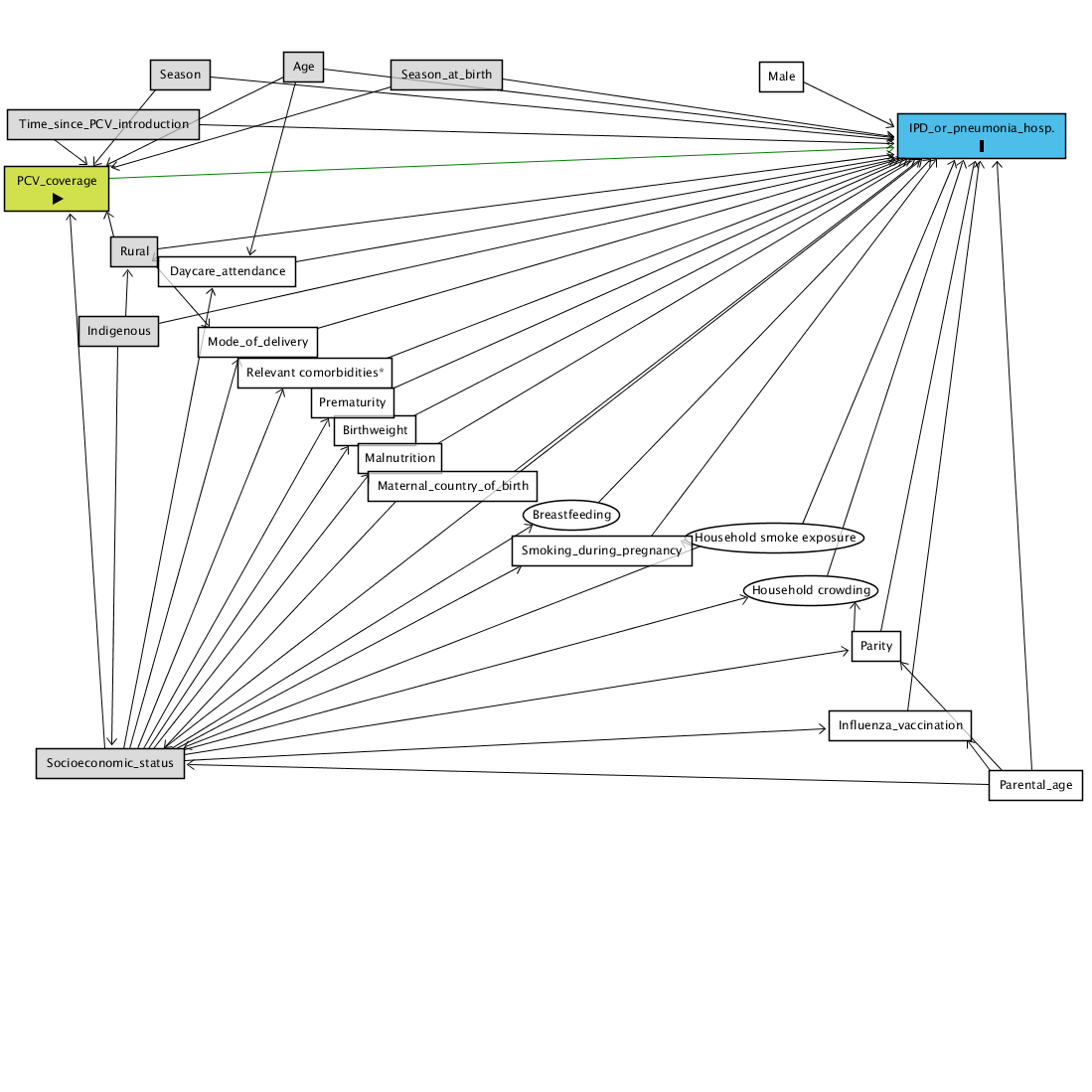


Figure 1: Directed acyclic graph of confounders for the invasive pneumococcal disease analysis; green box = exposure, blue box = outcome, grey boxes= variables for adjustment, white boxes= other variables, white circles = unmeasured variables; *Relevant comorbidities defined according to the immunisation handbook (category A and B conditions)

### Interactions

We are not aware of any specific interactions that should be accounted for in this analysis.

### Checking model fit

We will check model fit with the Pearson goodness of fit test. The fit will be considered inappropriate if the p-value is less than 0.05.

### Subgroups

We will conduct sub-analyses for the following groups if sample sizes allow:

- Aboriginal and Torres Strait Islander children, non-Aboriginal and Torres Strait Islander children
- By age group- <2 months, 2-23 months, 24-59 months

### Sensitivity analyses

To determine whether a higher SLA-level PCV coverage is required to indirect effects among completely un-vaccinated children compared to under-vaccinated children, we will conduct a sensitivity analysis in which children are censored at receipt of one dose of PCV.

# Dissemination plan

We are submitting or have submitted abstracts for the following conferences:

- 11th International Symposium on Pneumococci and Pneumococcal Diseases (ISPPD 2018)
- 16th Public Health Association of Australia (PHAA) National Immunisation Conference

We also plan to submit a paper to a peer-reviewed journal.

# References

1. National Centre for Immunisation Research and Surveillance (NCIRS). *History of vaccination in Australia*. 2018 2 April 2018]; Available from: <http://www.ncirs.edu.au/provider-resources/vaccination-history/>.

2. Loomis, D., D.B. Richardson, and L. Elliott, *Poisson regression analysis of ungrouped data.* Occup Environ Med, 2005. **62**(5): p. 325-9.

3. Pilishvili, T., et al., *Risk factors for invasive pneumococcal disease in children in the era of conjugate vaccine use.* Pediatrics, 2010. **126**(1): p. e9-17.

4. Wonodi, C.B., et al., *Evaluation of risk factors for severe pneumonia in children: the Pneumonia Etiology Research for Child Health study.* Clin Infect Dis, 2012. **54 Suppl 2**: p. S124-31.

5. Jardine, A., R.I. Menzies, and P.B. McIntyre, *Reduction in hospitalizations for pneumonia associated with the introduction of a pneumococcal conjugate vaccination schedule without a booster dose in Australia.* Pediatr Infect Dis J, 2010. **29**(7): p. 607-12.

# Appendices

**Appendix A: Flow-chart of construction of pneumococcal cohort born 1 January 2001 – 31 December 2012**

All live-born children, born to NSW or WA resident mothers, from 1 Jan 1996 to 31 Dec 2012, that have both RBDM and MDC records and were not on the ACIR exclude list:

N=1 953 877 (final.cohort_final)

Restricted to those born from 1 Jan 2001

N=1 407 181

Restricted to singletons only

N= 1 366 039 (97.1% of above)

Excluded children with any dose on or before DOB

N=1 365 904

Excluded where end date less than or equal to DOB

N=1 365 896

135 children with any dose on or before DOB excluded:

- 127 dose 1 before DOB
- 7 dose 2 before DOB
- 1 dose 3 before DOB

8 children excluded where end date less than or equal to DOB

Notification records for IPD for included children

N=1576 notifications from 1559 children (duplicates removed, keeping just the record with the earliest onset date)

ACIR records for included children for PRVNR, GNPNE, PRVTH, SYNFLX vaccine types

n= 3 081 900 records from 1 091 863 children i.e. 79.9% of children have one or more ACIR record

14 412 children from multiple births excluded

546 696 children born 1 Jan 1996 – 31 Dec 2000 excluded

**Appendix B: 10th Revision of the International Classification of Diseases Australian Modification (ICD-10-AM) codes for classification of pneumonia hospitalisation [5]**

| ICD-10-AM code | Description | AJ Groupings* |
| --- | --- | --- |
| J13 | Pneumococcal pneumonia | Pneumococcal pneumonia |
| J18.1 | Lobar pneumonia, unspecified |  |
| J10.0 | Influenza with pneumonia, influenza virus identified | Pneumonia due to other specified cause |
| J11.0 | Influenza with pneumonia, virus not identified |  |
| J12.0 | Pneumonia due to adenovirus |  |
| J12.1 | Pneumonia due to respiratory syncytial virus |  |
| J12.2 | Pneumonia due to parainfluenza virus |  |
| J12.8 | Pneumonia due to other virus not elsewhere classified |  |
| J12.9 | Viral pneumonia, unspecified |  |
| J15.0 | Pneumonia due to Klebsiella pneumoniae |  |
| J15.1 | Pneumonia due to Pseudomonas |  |
| J15.2 | Pneumonia due to Staphylococcus |  |
| J15.3 | Pneumonia due to Streptococcus, group B |  |
| J15.4 | Pneumonia due to other Streptococci |  |
| J15.5 | Pneumonia due to Escherichia coli |  |
| J15.6 | Pneumonia due to other aerobic Gram-negative bacteria |  |
| J15.8 | Other bacterial pneumonia |  |
| J15.7 | Pneumonia due to Mycoplasma pneumoniae |  |
| J15.9 | Bacterial pneumonia, unspecified |  |
| J16.0 | Chlamydial pneumonia |  |
| J16.8 | Pneumonia due to other specified infectious organisms |  |
| J17.0 | Pneumonia in bacterial diseases classified elsewhere |  |
| J17.1 | Pneumonia in viral diseases classified elsewhere |  |
| J17.2 | Pneumonia in mycoses |  |
| J17.3 | Pneumonia in parasitic diseases |  |
| J17.8 | Pneumonia in other infectious diseases classified elsewhere |  |
| B01.2 | Varicella pneumonia |  |
| B05.2 | Measles complicated by pneumonia |  |
| B37.1 | Pulmonary candidiasis |  |
| B59 | Pneumocystosis |  |
| J18.0 | Bronchopneumonia, unspecified | Pneumonia due to unspecified cause |
| J18.2 | Hypostatic pneumonia, unspecified |  |
| J18.8 | Other pneumonia, organism unspecified |  |
| J18.9 | Pneumonia, organism unspecified |  |
|  |  |  |

*Groupings according to Jardine et al. ^2^

**Appendix C: Alternative definitions for pneumococcal conjugate vaccine coverage**

|  |  |
| --- | --- |
| WHO survey method | Receipt of three doses (or two doses if country uses a 2+1 schedule) at 12-23 months of age |
| WHO administrative method | Receipt of three doses (or two doses if country uses a 2+1 schedule) among “surviving infants” – defined as number of children reaching their first birthday within a given year |
| Cohort method – used in Australia by NCIRS/Brynley Hull | For annual estimates - receipt of three doses among 12-month wide age cohort born in the previous year (.: at the date of assessment, children at 12-23 months of age)  For time trends - receipt of three, among a 3-month wide cohort – coverage assessment date 12 months after the last birth date of each cohort (.: at the date of assessment, children are 12-15 months of age) |
| Loughlin et al* | Number of children vaccinated among children <5 years of age – vaccinated defined as receiving two or more PCV doses for children <12 months. For children 12 months of age or older, a child is vaccinated if they received three PCV doses in the first year of life or at least one PCV dose after the age of 12 months |
| Grant et al* | Number of children fully immunized (age 7-12month: 3+ doses, age 12 to <16mo: 3+ doses <12 mo or 1+ dose >12 month, age 15-60 month: 1+ dose when received aged ≥12mo) among children < 5 years of age |

* To our knowledge, these are the only two studies investigating the same question of what PCV coverage is required for indirect effects. Note that in both settings there was a catch-up to <5 years of age (.: target age group <5 years of age). In Australia catch-up (for general population) was to <2 years of age.
