# Supplementary material for: Levels of pneumococcal conjugate vaccine coverage and indirect protection against invasive pneumococcal disease and pneumonia hospitalisations in Australia: An observational study
Source: PLoS Med. 2021 Aug 3;18(8):e1003733. doi: 10.1371/journal.pmed.1003733 (PMC8376256; doi:10.1371/journal.pmed.1003733)
Supplement: Attachment — Submitted filename: PCVherdeffects_PLOSMed_response.docx [file pmed.1003733.s004.docx]

Wednesday, 26 May 2021

Dear Dr Raffaella Bosurgi,

Thank you for giving us the opportunity to respond to the reviewers’ comments. Please find our point-by-point response below.

**Requests from the editors:**

1.Title: Please revise your title according to PLOS Medicine's style. Your title must be nondeclarative and not a question. It should begin with main concept if possible. "Effect of" should be used only if causality can be inferred, i.e., for an RCT. Please place the study design ("A randomized controlled trial," "A retrospective study," "A modelling study," etc.) in the subtitle (ie, after a colon).

**Authors’ response:** Thank you – we have revised the title to: “Pneumococcal conjugate vaccine coverage and indirect protection against invasive pneumococcal disease and pneumonia hospitalisations in Australia: a retrospective record linkage cohort study”

2. Financial disclosure: Please include the statement “The funders had no role in study design, data collection and analysis, decision to publish, or preparation of the manuscript” unless this does not apply.

**Authors’ response:** Thank you – we have added this statement under the ‘Funding’ subheading at the end of the manuscript.

3. Abstract: Please structure your abstract using the PLOS Medicine headings (Background, Methods and Findings, Conclusions).

**Authors’ response:** Thank you – we have amended the abstract headings in line with journal requirements.

4. Abstract: Methods and Findings: Please indicate in the abstract which two states of Australia are included.

**Authors’ response**: Thank you – we have added the two states to the abstract (New South Wales and Western Australia).

5. Abstract: Methods and Findings: Please include the important variables that are adjusted for in the analyses.

**Authors’ response:** We understand that the variables selected for adjustment are critical, however there are too many variables to list within the abstract due to the word count restrictions. Instead, we have added a sentence to explain our methods for variable selection. “Potential confounding variables were selected for adjustment *a priori* with the assistance of a directed acyclic graph.”

The full list of variables that are adjusted for in the analyses are described in the methods. These were age group, calendar year, Indigenous status, season, Index of Relative Socio-economic Advantage and Disadvantage (IRSAD) score, Accessibility or Remoteness Index of Australia (ARIA) category, birth weight, gestational age, maternal smoking during pregnancy, number of previous pregnancies, and previous hospitalisation with an ICD code corresponding to the presence of a medical condition increasing risk of IPD.

6. Abstract: Methods and Findings: Please quantify the main results (with 95% CIs and p values).

**Authors’ response**: Thank you – we have quantified the main results in the abstracts.

“There were strong inverse associations between PCV coverage and the incidence of PCV7-type IPD (adjusted incidence rate ratio [aIRR] 0.967, 95% CI 0.958-0.975, p-value <0.001), and pneumonia hospitalisations (all-cause pneumonia: aIRR 0.991 95% CI 0.990-0.994, p-value<0.001) among under-vaccinated children.”

7. Abstract: Methods and Findings: In the last sentence of the Abstract Methods and Findings section, please describe the main limitation(s) of the study's methodology.

**Authors’ response:** We have added an additional sentence to describe the study’s main limitations. “The main limitations of this study include the potential for differential loss to follow-up, geographical misclassification of children (based on addressed at birth only) and unmeasured confounders.”

8. Abstract: Conclusions: Please address the study implications without overreaching what can be concluded from the data; the phrase "In this study, we observed ..." may be useful.

**Authors’ response:** We have amended the abstract conclusions to read: “In this study we observed substantial indirect protection at low PCV coverage, challenging assumptions high vaccine coverage is required.”

9. Author Summary: At this stage, we ask that you include a short, non-technical Author Summary of your research to make findings accessible to a wide audience that includes both scientists and non-scientists. The Author Summary should immediately follow the Abstract in your revised manuscript. This text is subject to editorial change and should be distinct from the scientific abstract. Please see our author guidelines for more information: <https://journals.plos.org/plosmedicine/s/revising-your-manuscript#loc-author-summary>

**Authors’ response:** We have added an author summary section:

“**Author summary**

Why was this study done?

- Pneumococcal conjugate vaccines (PCVs) reduce the burden of pneumococcal disease in vaccinated and unvaccinated populations through both direct and indirect (herd) effects.
- The indirect effects comprise a substantial component of overall vaccine impact, contributing to the cost-effectiveness of the vaccine but little is known about what factors contribute to herd protection, including vaccination coverage.
- In this study, we examined association between PCV coverage and indirect effects within in diverse populations within Australia

What did the researchers do and find?

- Using a large dataset of 1.3 million children from two states in Australia, we quantified the relationship between PCV coverage within small geographical units and indirect protection against pneumococcal disease. We also performed similar analyses for infants too young to be fully vaccinated, urban, rural and Indigenous populations.
- There were strong inverse relationships between PCV coverage and the incidence of severe invasive disease due to vaccine types and pneumonia hospitalisations among under-vaccinated children i.e. higher coverage were associated with greater reductions in disease due to indirect effects. We also found substantial indirect effects at relatively low levels of PCV coverage. We estimated that 50% and 90% coverage of 7-valent PCV (PCV7) among children under five years of age prevented almost three-quarters (72.5%, 95% CI 51.6-84.4) and almost all (95.2%, 95% CI 89.4-97.8) of PCV7-type severe invasive disease, respectively. For pneumonia, we estimated that 50% and 90% coverage was sufficient to prevent one-third (33.3%, 95% CI 27.3-38.8) and about half (51.7%, 95% CI 43.7-58.6) of all-cause pneumonia hospitalisations among under-vaccinated children.
- These trends were similar for children less than four months old, urban, rural and Indigenous populations, although these effects were smaller for rural and Indigenous populations. There was also a trend towards decreasing incidence of PCV13-type IPD among under-vaccinated children as PCV13 coverage increased.

What do these findings mean?

- Our results challenge existing assumptions that high PCV coverage is required to achieve substantial indirect protection.
- Understanding the determinants of indirect effects are particularly urgent as countries that have controlled vaccine-type pneumococcal disease consider using reducing the number of PCV doses (from three to two). Reduced dose schedules have the potential to significantly lower program costs while maintaining vaccine impact, providing indirect protection is achieved and preserved. “

10. Main text: Please use square brackets for in-text citations, like this [1]. For citations of multiple references, please do not include spaces within brackets.

**Authors’ response:** Thank you – we have updated the citation style throughout the manuscript.

11. Main text: Please include line numbers with the revised version.

**Authors’ response:** Thank you – we have included line numbers with the revised version.

12. Introduction: Paragraph 2: Please remove the trademark symbol from Prevnar, PNEUMOSIL, and Synflorix.

**Authors’ response:** Thank you – we have removed the trademark symbols.

13. Methods: Please ensure that the study is reported according to the STROBE guideline, and include the completed STROBE checklist as Supporting Information. Please add the following statement, or similar, to the Methods: "This study is reported as per the Strengthening the Reporting of Observational Studies in Epidemiology (STROBE) guideline (S1 Checklist)."

The STROBE guideline can be found here: [http://www.equator-network.org/reporting-guidelines/strobe/](https://protect-au.mimecast.com/s/LhWdC2xMxLhV0x5GS66R2c?domain=equator-network.org)

When completing the checklist, please use section and paragraph numbers, rather than page numbers.

**Authors’ response:** Thank you – we have completed and attached the RECORD checklist – an extension of the STROBE checklist for studies conducted using routinely-collected health data.

14. Methods: Did your study have a prospective protocol or analysis plan? Please state this (either way) early in the Methods section.

a) If a prospective analysis plan (from your funding proposal, IRB or other ethics committee submission, study protocol, or other planning document written before analyzing the data) was used in designing the study, please include the relevant prospectively written document with your revised manuscript as a Supporting Information file to be published alongside your study, and cite it in the Methods section. A legend for this file should be included at the end of your manuscript. 

b) If no such document exists, please make sure that the Methods section transparently describes when analyses were planned, and when/why any data-driven changes to analyses took place. 

c) In either case, changes in the analysis-- including those made in response to peer review comments-- should be identified as such in the Methods section of the paper, with rationale.

**Authors’ response:** Thank you – our study included a prospective data analysis plan (DAP). We have added this to this Methods section and included the DAP as a Supporting Information File. There were no data driven changes to our final analyses, compared to our prospective DAP.

15. Method: “Infant pneumococcal vaccination in Australia” Please consider whether this section may be better incorporated into the Introduction.

**Authors’ response:** Thank you – we have moved the paragraph titled “Infant pneumococcal vaccination in Australia” to the introduction.

16. Results: Please present a table of summary demographic characteristics for the cohort.

Authors’ response: Thank you – we have added a table of summary demographic characteristics for the cohort.

Table 1: Cohort characteristics by Indigenous status, New South Wales and Western Australia, 2001-2012

|  |  | Non-Indigenous,  n (%), N=1 299 409 | Indigenous,  n(%), N=66 484 |
| --- | --- | --- | --- |
| State of birth registration | New South Wales | 993 936 (76.5) | 45 551 (68.5) |
|  | Western Australia | 305 473 (25.5) | 20 933 (31.5) |
| Sex | Male | 668 021 (95.1) | 34 284 (4.9) |
|  | Female | 631 388 (95.2) | 32 200 (4.9) |
| Socioeconomic index | 91-100% (least disadvantaged) | 160 519 (12.4) | 990 (1.5) |
|  | 76-90% | 199 055 (15.3) | 2 739 (4.1) |
|  | 26-75% | 582 833 (44.9) | 21 704 (32.7) |
|  | 11-75% | 196 074 (15.1) | 15 371 (23.1) |
|  | 0-10% (most disadvantaged) | 126 124 (9.7) | 21 996 (33.1) |
|  | Missing | 34 804 (2.7) | 3 684 (5.5) |
| Remoteness index | Major cities (urban) | 989 470 (76.2) | 27 633 (42.6) |
|  | Inner regional (urban) | 189 454 (14.6) | 17 246 (25.9) |
|  | Outer regional (rural) | 71 056 (5.5) | 10 899 (16.4) |
|  | Remote (rural) | 13 681 (1.1) | 4 813 (7.2) |
|  | Very remote (rural) | 3 310 (0.3) | 2 711 (4.8) |
|  | Missing | 32 438 (2.5) | 3 182 (4.8) |
| Number of previous pregnancies (parity) | 0 | 520 276 (40.0) | 22 119 (33.3) |
|  | 1 | 434 073 (33.4) | 16 512 (24.8) |
|  | 2 | 205 396 (15.8) | 11 002 (16.6) |
|  | ≥ 3 | 138 257 (10.6) | 16 786 (25.3) |
| Birthweight (g) | <1500 | 7 105 (0.6) | 833 (1.3) |
|  | 1500-2499 | 45 846 (3.5) | 5 417 (8.2) |
|  | 2500-3499 | 668 358 (51.4) | 36 029 (54.2) |
|  | 3500-4499 | 555 005 (42.7) | 23 126 (34.8) |
|  | ≥4500 | 22 771 (1.8) | 1 065 (1.6) |
|  | Missing | 324 (0.0) | 14 (0.0) |
| Prematurity (gestational age < 33 weeks) | Preterm | 8 539 (0.7) | 993 (1.5) |
|  | Missing | 156 (0.0) | 30 (0.1) |
| Parental smoking | Yes | 151 309 (11.6) | 30 309 (45.6) |
|  | Missing | 2890 (0.2) | 81 (0.1) |
| Hospital admission aged < 6 weeks | | 193 020 (14.9) | 13 566 (20.4) |
| Any hospital admission for risk condition | | 47 705 (3.7) | 3 382 (5.1) |

17. Results: Please provide both 95% CIs and p values wherever relevant alongside results presented in the text; for example, when describing the associations between PCV coverage and PCV7 and PCV13-type IPV/pneumonia incidence.

**Authors’ response:** Thank you – we have reviewed the document to ensure that 95% CIs and p-values are presented alongside results in the text where appropriate.

18. Discussion: Please present and organize the Discussion as follows: a short, clear summary of the article's findings; what the study adds to existing research and where and why the results may differ from previous research; strengths and limitations of the study; implications and next steps for research, clinical practice, and/or public policy; one-paragraph conclusion.

**Authors’ response:** Thank you - we agree with the editor's suggested discussion format. We believe that our discussion broadly follows this format.

- Paragraphs 1-5 – each paragraph describe a key finding and discusses it in relation to previous research
- Paragraph 6 – study strengths and limitations
- Paragraph 7 and 8 – implications and next steps for research and policy

19. References: Please use the "Vancouver" style for reference formatting, and see our website for other reference guidelines <https://journals.plos.org/plosmedicine/s/submission-guidelines#loc-references>

**Authors’ response:** Thank you – we have updated the citation style throughout the manuscript.

20. Figures 4, 5, and 7: please indicate in the legends that the blue shaded area represents the 95% confidence interval.

**Authors’ response:** Thank you - we have added an explanation of the blue shaded areas representing 95% confidence intervals to the figure captions.

**Comments from the reviewers:**

**Reviewer #1:** The article by Chan et al describes an assessment of indirect protection derived from vaccination of children with pneumococcal conjugate vaccines in two States in Australia. Using a large research dataset linking birth certificates, vaccination records, laboratory surveillance results, and hospitalization data, investigators designed a retrospective cohort study to examine the incidence of invasive pneumococcal disease and pneumonia hospitalizations among children younger than 5 years. Incidence estimates were compared across different vaccination period, based on vaccine formulations and vaccination schedules. Incidence estimates were generated separately for all children and for under-vaccinated children, and assessments among under-vaccinated children were used to investigate the incidence of study outcomes by area vaccinate coverage estimates for an assessment of indirect protection. The investigators report a substantial indirect protection among under-vaccinated children derived from vaccination of young children with pneumococcal conjugate vaccines, with declining disease incidence with increasing vaccination coverage in the children's resident area. Material reductions were reported even with vaccination coverage of ~50%, and substantial benefits were calculated based on the estimated benefit of vaccination. 

Understanding the critical coverage of vaccination needed to attain population level indirect protection is of great public health interest. Yet, this is rarely examined using actual individual level data. These assessments are particularly important for expensive vaccines such as pneumococcal conjugate vaccines. So, this is a very important and relevant study. Linking multiple available databases, investigators have designed a powerful research data platform to address the questions of interest. The article is very well written, and the assessments are clearly described. This is a very elegant study with very important public health implications. However, I have a few comments/questions:

It is unclear how losses to follow-up were identified in the retrospective cohort study. This can be problematic if children leaving the study area were less likely to complete vaccination and if their outcomes were also not recorded in the source databases. Per the description in the article, based on birth certificates - everyone identified was assumed to remain under observation, but I am not sure this was actually demonstrated or discussed. It would be useful to conduct an exploration of an unrelated outcome (i.e. unlikely to be affected by vaccination) to ensure differential losses to follow-up were not a problem in the study. Alternative sources of follow-up information from other regions of the country could be useful as well. This potential issue should be addressed and discussed.

**Authors’ response:** Thank you for raising an important point regarding loss to follow-up. We have added additional section to the discussion about this important study limitation.

“Lastly, an important limitation of this study is the potential for loss to follow-up in children who have moved interstate (missing outcome data) or overseas (missing vaccination and outcome data). From 2001 to 2012, the annual interstate out-migration of children less than five years of age ranged from 1.6-2.2% in New South Wales and 1.4-2.1% in Western Australia (Australian Bureau of Statistics). Data available from 2004-2012 indicates that overseas out-migration was less common, ranging from 0.9-1.2% (Australian Bureau of Statistics). Since our analyses are for children up to five years of age, we estimate that the cumulative unobserved loss to follow-up time to be less than 15% - in line with recommendations for cohort studies (Kristman, European Journal of Epidemiology, 2004). Furthermore, we do not expect differential loss to follow-up between vaccinated and under-vaccinated children or as vaccine coverage rises over time, since migration patterns have remained constant.”

It would be very valuable to provide some additional description and references to substantiate the validity of the vaccination registry data - if vaccinated children somehow had their vaccination records affected they may appear as under-vaccinated when they were indeed fully vaccinated. This important misclassification possibility should be discussed in more detail as well.

**Authors’ response:** Thank you for the suggestion – we agree that providing a description of the validity of the vaccination registry data is important. We have added the following sentence to the discussion.

“We consider the risk of misclassification of vaccination status to be low, since vaccination status was determined using an immunisation registry, which has previously been demonstrated to underestimate coverage by less than 5% (Hull, ANZJPH, 2007).”

Previous related studies (see work by Loughlin et al [ref 30] and by Hammit et al. Lancet Global Health 2014) have estimated that approximately 70% coverage would be needed to attain indirect protection against carriage of vaccine types. The apparent discrepancy is intriguing as carriage is the recognized precursor for pneumococcal diseases. Reconciling those previous findings with the current estimates would be a valuable addition to the discussion section.

**Authors’ response:** Thank you – we agree that reconciling our estimates with previous studies by Loughlin et al would be a valuable addition to the discussion section. We have added and amended the following paragraph:

“However, in contrast to previous pneumococcal carriage studies of PCV10 and 13, which reported indirect effects at 58-75% coverage, we identified substantial indirect effects at lower rates of PCV7 coverage (Hammitt, Lancet GH, 2014; Loughlin, PIDJ, 2014; Grant, PIDJ). While not directly comparable, the indirect effect of PCV against disease can be expected to reflect indirect effects on carriage, since these effects are mediated by reductions in carriage. The variation in results may be due to differences in analytic method. Compared to annual carriage surveys conducted by Hammitt et al., we analysed at quarterly intervals allowing us to detect indirect effects at lower coverage rates. Grant et al. defined indirect effects as a statistically significant reduction in vaccine-type carriage prevalence, a measure which depends on both the effect size and sample size. While Loughlin et al. chose to define indirect effects *a priori* as a 50% decline in vaccine-type carriage. By modelling the association, we were able to quantify degree of indirect effects at varying coverage rates, facilitating future comparisons between studies with varying sample sizes and methods. Differences in the relationship between PCV coverage and indirect effects between studies may also be due to differences in the vaccine type studied (i.e. PCV7, PCV10 or PCV13) or context.”

“While our analyses of PCV7-type IPD showed the steep declines in disease, especially up to 50% PCV coverage, we observed flatter associations between PCV coverage and both PCV13-type IPD and all-cause pneumonia hospitalisations. . These results are consistent with previous studies indicating higher coverage rates (58-75) were required for substantial indirect effects using PCV10 and PCV13. (Hammitt, Lancet GH, 2014; Loughlin, PIDJ, 2014; Grant, PIDJ). This may be explained by previous research indicating lower vaccine effectiveness against the additional serotypes in PCV13 (Gessner, Vaccine, 2019; Waight, Lancet ID, 2015).”

The relatively limited impact of vaccination coverage in rural and indigenous populations is very intriguing and needs more discussion. The lack of significant associations among younger infants is intriguing as well. In part, these observations were attributed to limited social mixing in rural population relative to urban populations. However, ref [21] suggest there are more intense contacts in rural indigenous settings (e.g. facilitated by crowding and other factors) which facilitates spread of infections. The discussion needs to clarify and reconcile these elements. Also, the sample sizes were relatively small for some groups, but the rural subgroup was not that small. So other factors, separate from limited statistical precision, may potentially play a role in the current findings.

**Authors’ response:** Thank you for the opportunity to further discuss the limited impact of PCV coverage among rural and Indigenous populations. Firstly, we have amended the Figure 1 to better highlight earlier introduction of PCV for Indigenous children, in June 2001 shortly after the start of our study period. Hence, under-vaccinated Indigenous children were already benefiting from some degree of indirect effects at the start of our study’s observation period. This likely explains the unexpectedly low modelled incidence of PCV7-type among under-vaccinated Indigenous children at zero coverage despite previous research indicating more intense contacts in rural Indigenous settings. We have amended the discussion to highlight the impact of this on our ability to detect the full indirect effects of PCV introduction in this population, given the lack of baseline data.

Figure 1: Quarterly coverage* of any pneumococcal conjugate vaccine (PCV) and 13-valent PCV (PCV13) among Indigenous and all children under five years of age, New South Wales and Western Australia, 2001-2012

“Importantly we found similar trends among rural populations and Indigenous populations, although the baseline rates of IPD were lower, effect sizes were smaller and confidence intervals were wide, which likely reflects the smaller sample size available for analysis. These findings are important as they reflect indirect effects in different settings which are likely to have different patterns of social mixing and therefore patterns of pneumococcal disease transmission. The modelled incidence of PCV7-type among under-vaccinated Indigenous children at zero coverage was lower than expected, given Indigenous children are known to be at high risk of pneumococcal disease. This is likely due to the earlier introduction of PCV for Indigenous children in 2001, which meant under-vaccinated Indigenous children were already benefiting from some degree of indirect effects at the start of our study’s observation period. Without sufficient baseline data prior to vaccine introduction, our analyses likely underestimate the full extent of indirect effects among Indigenous children. Given that Indigenous children make up 17.3% of our rural cohort, the earlier introduction of PCV among Indigenous children may also have impacted on our estimates of indirect effects in rural settings. However, the low baseline rates of disease may also be attributable to a more sparsely populated rural population with less social mixing compared to urban settings. Previous studies on the direct effect of PCV, found the vaccine to be equally effective among Indigenous children and children from rural areas, suggesting that our results are not due to reduced vaccine effectiveness in these groups. (Fathima, Lancet CAH, 2019; Gidding, Vaccine, 2018)”

Some covariates appear to be listed in the DAG figure, but it is not clear if those were accounted for in the regression analyses (e.g. number of children/siblings in the house, flu vaccination, etc.). Please reconcile the narratives and clarify/revise accordingly.

**Authors’ response:** Thank you – we have clarified this in the supplementary materials (page 6):

“We identified the following potential confounders for adjustment: age, season, influenza vaccination, socioeconomic status, remoteness, low birth weight, prematurity, parental smoking, number of siblings and high risk comorbidity. The study variables that we used as proxies for the potential confounders identified are listed in supplementary Table 2. In most cases, there was a direct correlation between potential variables listed in the DAG and study variables used for adjustment, with the exception of number of siblings – for which we used parity as a proxy since this variable was not available. Influenza vaccination status was initially investigated by excluded from final models due to small numbers of recorded doses and collinearity with PCV.”

**Reviewer #2:** This retrospective population-based cohort study, comprising a subset of children under five years of age from a dataset of all children born in New South Wales (NSW) and Western Australia (WA) between 2001 and 2012, investigates the association between population PCV coverage and indirect protection against invasive pneumococcal disease and pneumonia hospitalisations among under-vaccinated Australian children.

Comments:

"To visualise any geographic heterogeneity in PCV coverage, we mapped coverage within Statistical Local Areas among children under five years of age at three time points - December 2005, December 2009, December 2012"
Can the authors please add an explanation here to clarify why these time points were chosen to map coverage?

**Authors’ response:** Thank you for this question. We chose the three time periods to represent the early post-PCV7 period, the late post-PCV7 period (when indirect effects have likely to have matured and serotype replacement with non-PCV7 serotypes are likely to peak prior to PCV13 introduction) and the early PCV13 period (to determine the effects of PCV13 on the additional PCV13, non-PCV7 serotypes).We have amended the sentence to clarify why these time points were chosen:

"To visualise any geographic heterogeneity in PCV coverage, we mapped coverage within Statistical Local Areas among children under five years of age at three time points - December 2005, December 2009 and December 2012. The three time periods were chosen to represent the early post-PCV7 period, the late post-PCV7 period and the early PCV13 period.”

The use of Poisson regression models is technically appropriate, and the inclusion of various potential confounding variables is robust. Furthermore, suitable subgroup and sensitivity analyses have been included in the study.

Typo in: " Person-time for each child started at birth and was censored at the earliest of the following: death, when the child reached five years of age or at the end of the of the study period."

**Authors’ response:** Thank you – we have deleted the additional “of the” in the sentence.

Figure 4 typo: A and B (currently both labelled as B)

**Authors’ response:** Thank you – we have re-labelled the figures A and B.

Overall, this is a very well written report within which the main study limitations have been fairly acknowledged. It also comes with an extensive and thorough analysis contained within the supplementary material.

**Authors’ response:** Thank you – we appreciate the positive feedback!

**Reviewer #3:** Substantial indirect protection against invasive pneumococcal disease and pneumonia
hospitalisations in Australian children at low levels of pneumococcal conjugate vaccine
coverage: an observational study

This study used IPD incidence and serotype data from state-based passive surveillance, Vaccine status from Australian Childhood Immunisation Registry and Hospitalisation data covering all inpatient separations to calculate the decline in VST disease, PCV7 or PCV13 compared to PCV7 and PCV13 uptake. Their strategy was to calculate the incidence of each disease outcome during the person-time for which children were under-vaccinated up to five years of age. They divided each child's under-vaccinated person-time into successive three-month intervals and linked these intervals to PCV coverage in their SLA of residence during the same time period. Thirty one percent of the childfen were among under-vaccinated or unvaccinated children, the majority of whom were unvaccinated. The conventional wisdom has been that reduction in colonization with VST Sp and indirect effect was observed beginning at ~ 50% vaccine penetration and increased with increasing vaccine uptake. The authors find lower level of community uptake, specifically in urban centers, has a larger impact than previously identified. If correct, this would have important implications as high uptake, especially with dose after 12 months of age, may be more difficulty to achieve in certain setting.

Table 1 reports the declining incidence in immunized and unimmunized populations over intervals of time and the subsequent graph shows the dramatic increasing in vaccine uptake occurring over a few year. The decline per percent increase in vaccine uptake is then calculated. I found myself wanting to see more granular data; the incidence decline from 7.3 to 1.3 over a 11 year time line. I think showing the decline each year from 2001 through 2011 and the coverage each year for each group would improve clarity for the reader to view the uptake in the community and the decline in the vaccinated and under-vaccinated group. The rapid uptake as demonstrated in figure 3 does not allow the impact of the incremental uptake after introduction to be assessed as the data is presented for the 5 year period.

**Authors’ response:** Thank you for your suggestion. We agree that presenting more granular data on disease incidence and vaccine uptake in the same table would be useful. We have added this table to S1 File, Tables 8 and 9. We have also clarified that Figure 1 presents quarterly PCV coverage which captures the rapid increase in coverage shortly after vaccine introduction.

## Table 8: Annual pneumococcal conjugate vaccine coverage (PCV) and rate of 7-valent PCV-type invasive pneumococcal disease (IPD) among all children and under-vaccinated children and among children under five years of age, New South Wales and Western Australia, 2005-2012

| Year | All children | | Under-vaccinated children | | Any PCV coverage, median (IQR) |
| --- | --- | --- | --- | --- | --- |
|  | **Cases/person-years** | **PCV7-type IPD rate** | **Cases/person years** | **PCV7-type IPD rate** |  |
| 2001 | 3/52 599 | 0.6 (0.2-1.8) | 3/52 537 | 0.6 (0.2-1.8) | - |
| 2002 | 98/ 156 546 | 6.3 (5.1-7.6) | 98/154 962 | 6.3 (5.2-7.7) | - |
| 2003 | 157/ 260 627 | 6.0 (5.2-7.0) | 155/254 963 | 6.1 (5.2-7.1) | - |
| 2004 | 202/365 561 | 5.3 (4.8-6.3) | 199/349 455 | 5.7 (5.0-6.5) | - |
| 2005 | 82/472 529 | 1.7 (1.4-2.2) | 76/322 142 | 2.4 (1.9-3.0) | 17∙5 (15∙8-19∙2) |
| 2006 | 25/532 325 | 0.5 (0.3-0.7) | 16/238 373 | 0.7 (0.4-1.1) | 27∙2 (24∙7-29∙2) |
| 2007 | 14/545 472 | 0.3 (0.2-0.4) | 4/150 083 | 0.3 (0.1-0.7) | 38∙1 (35∙6-40∙3) |
| 2008 | 12/560 578 | 0.2 (0.1-0.4) | 3/90 950 | 0.3 (0.1-1.0) | 46∙1 (43∙0-48∙1) |
| 2009 | 7/575 653 | 0.1 (0.1-0.3) | 3/72 796 | 0.4 (0.1-1.3) | 53∙5 (50∙7-55∙9) |
| 2010 | 4/590 098 | 0.1 (0.0-0.2) | 1/70 675 | 0.1 (0.0-1.0) | 62∙0 (59∙1-63∙9) |
| 2011 | 5/598 864 | 0.1 (0.0-0.2) | 2/70 602 | 0.3 (0.1-1.1) | 72∙4 (69∙7-74∙5) |
| 2012 | 5/602 610 | 0.1 (0.0-0.2) | 0/71 514 | 0.0 | 86∙8 (84∙5-88∙9) |

*Calculated as of 31 December of each year

## Table 9: Annual 13-valent pneumococcal conjugate vaccine coverage (PCV13) and rate of PCV13, non PCV7-type invasive pneumococcal disease (IPD) among all children and under-vaccinated children and among children under five years of age, New South Wales and Western Australia, 2005-2012

| Year | All children | | Under-vaccinated children | | PCV13 coverage, median (IQR) |
| --- | --- | --- | --- | --- | --- |
|  | **Cases/person-years** | **PCV7-type IPD rate** | **Cases/person years** | **PCV7-type IPD rate** |  |
| 2001 | 0/52 600 | 0.0 | 0/52 600 | 0.0 | - |
| 2002 | 5/156 588 | 0.3 (0.1-0.8) | 5/156 588 | 0.3 (0.1-0.8) | - |
| 2003 | 6/260 790 | 0.2 (0.1-0.5) | 6/260 791 | 0.2 (0.1-0.5) | - |
| 2004 | 18/365 890 | 0.5 (0.3-0.8) | 18/365 890 | 0.5 (0.3-0.8) | - |
| 2005 | 29/472 984 | 0.6 (0.4-0.9) | 29/472 984 | 0.6 (0.4-0.9) | - |
| 2006 | 19/532 730 | 0.4 (0.2-0.6) | 19/532 730 | 0.4 (0.2-0.6) | - |
| 2007 | 38/545 702 | 0.7 (0.5-1.0) | 38/545 700 | 0.7 (0.5-1.0) | - |
| 2008 | 59/560 632 | 1.0 (0.8-1.4) | 59/560 622 | 1.1 (0.8-1.4) | - |
| 2009 | 54/575 567 | 0.9 (0.7-1.2) | 54/575 539 | 0.9 (0.7-1.2) | - |
| 2010 | 66/589 960 | 1.1 (0.9-1.4) | 66/589 869 | 1.1 (0.9-1.4) | - |
| 2011 | 62/598 699 | 1.0 (0.8-1.3) | 62/584 674 | 1.1 (0.8-1.4) | 6∙8 (5∙6-8∙1) |
| 2012 | 32/602 458 | 0.5 (0.4-0.8) | 28/430 478 | 0.7 (0.4-0.9) | 32∙1 (29∙1-35∙4) |

As vaccine uptake included a catch up through two years it is unclear if the age distribution in the comparison groups are comparable for each 3 month block assessed. Obviously there is an age specific risk for IPD and pneumonia and if the age distribution of each group differs then the incidence within those group may differ regardless of the vaccine impact. Is it possible the vaccinated group is likely biased toward children under 3, while the unvaccinated is biased toward children over 2 which would be a lower risk for IPD?

**Authors’ response:** We agree with your assessment and consider age to be an important confounder in this analysis. We put considerable thought into how we might adjust for differences in the age distribution of vaccinated and under-vaccinated children over time. In the end, we opted to categorise each block of person-time into five age groups (0-11 months, 12-23 months, 24-35 months, 36-47 months and 48-59 months) and adjust as a categorical variable for each of our analyses (S1 File Table 1 and Figure 2). We chose to adjust for age as a categorical variable rather than a continuous variable because the relationship between IPD and age is non-linear, with IPD typically peaking at 12-23 months age among children under five in Australia (Toms, Communicable Diseases Intelligence, 2016).

The graphs of vaccine uptake vs decline in IPD (figure 4 and 5) demonstrate substantially different relationships for PCV7 uptake and decline compared to PCV13 and decline. The authors explain this by difference in effectiveness against the PCV7 vs the PCV13 unique serotypes. As the vaccine is less effective against serotype 3, it would be of value to compare with and without serotype 3 (for PCV13) as a sensitivity analysis to determine if the concept that 40% penetration is sufficient to produce substantial indirect benefit is limited to PCV7 or more broadly applicable to pneumococcal conjugate vaccines. And if limited to PCV7, why???

**Authors’ response:** Thank you for you’re the opportunity to further consider the substantially different results for PCV13. We agree that a sensitivity analysis excluding serotype 3 from our PCV13 analyses is warranted. However, upon review of our data, we identified only nine cases of serotype 3, comprising only 2.2% of PCV13- non-PCV7-type IPD across the study period. Therefore, we elected not to perform a sensitivity analysis as we do not believe it would yield significantly different results.

However, we have amended our results and discussion to highlight the limited period of monitoring post-PCV13 introduction available for our analysis, which is likely to impede our ability to detect the full indirect effects of PCV13 introduction. Given PCV13 coverage only reached a median of 32.1% at the end of our study period, we have amended Figure 4 to avoid predicting any declines in the rates of PCV13- non-PCV7-type IPD beyond 40% coverage.

Figure 4: Estimated rate of vaccine-type invasive pneumococcal disease incidence* and 95% confidence intervals (blue shading) among under-vaccinated children under five years, at each decile of pneumococcal conjugate vaccine (PCV) coverage, New South Wales and Western Australia, 2001-12. *PCV13, non-PCV7-type IPD incidence was only estimated up to 40% as there were few data points available

“While our analyses of PCV7-type IPD showed the steep declines in disease, especially up to 50% PCV coverage, we observed flatter associations between PCV coverage and both PCV13-type IPD and all-cause pneumonia hospitalisations. These results are also consistent with previous studies indicating higher coverage rates (58-75%) are required for indirect effects using of PCV10 and PCV13. (Hammitt, Lancet GH, 2014; Loughlin, PIDJ, 2014; Grant, PIDJ). This may be explained by previous research indicating lower vaccine effectiveness against the additional serotypes in PCV13. However, there is a high degree of uncertainty around our PCV13 estimates since we have limited data post-PCV13 introduction (1.5 years) and a small number of PCV13, non-PCV7-type IPD cases. At the end of our study period, PCV13 coverage had only reached a median 32.1% among children under five. Therefore, our analyses are unable to capture the full indirect effects of PCV13, which are likely to increase as vaccine coverage rises over time.”

Figure 5 demonstrates that different populations have different dynamics. The indirect effect for those less than 4 months and Indigenous and rural individuals has a different relationship between vaccine uptake and indirect effect compared to urban environment. How does the disease epidemiology impact on this relationship? It appears that incidence in first 4 month is relatively low; that urban children have highest incidence; and Indigenous children are not as high as expected as I thought their rate of disease was highest in the population?? Are these relationships between vaccine uptake and disease decline similar for direct effect or only for indirect effect?

**Authors’ response:** Thank you for the opportunity to expand on the relevance of disease epidemiology to the relationship between PCV coverage and indirect effects. Firstly, we have amended the Figure 1 to better highlight earlier introduction of PCV for Indigenous children, in June 2001 shortly after the start of our study period. We have also amended the discussion to highlight the impact of this on our ability to detect the full indirect effects of PCV introduction in this population, given the lack of baseline data.

Figure 1: Quarterly coverage* of any pneumococcal conjugate vaccine (PCV) and 13-valent PCV (PCV13) among Indigenous and all children under five years of age, New South Wales and Western Australia, 2001-2012

“Importantly, we found similar trends among rural populations and Indigenous populations, although these effects were smaller and had large confidence intervals which likely reflects the smaller sample size available for analysis. These findings are important as they reflect indirect effects in different settings with different patterns of social mixing and therefore patterns of pneumococcal disease transmission. The modelled incidence of PCV7-type disease at zero coverage was lower than expected, given Indigenous children are known to be at high risk of pneumococcal disease. This is likely due to the earlier introduction of PCV for Indigenous children in 2001, which meant under-vaccinated Indigenous children were already benefiting from some degree of indirect effects at the start of our study’s observation period. Without sufficient baseline data prior to vaccine introduction, our analyses likely underestimate the full extent of indirect effects among Indigenous children. Given that Indigenous children make up 17.3% of our rural cohort, the earlier introduction of PCV among Indigenous children may also have impacted on our estimates of indirect effects in rural settings. However, the low baseline rates of disease may also be attributable to a more Ssparsely populated rural populations are likely to have with less social mixing compared to urban settings .[31]. Previous studies on the direct effect of PCV, found the vaccine to be equally effective among Indigenous children and children from rural areas, suggesting that our results are not due to reduced vaccine effectiveness in these groups. (Fathima, Lancet CAH, 2019; Gidding, Vaccine, 2018)”

Other comments:

The UK introduction of 1+1 regimens was after nearly 10 years of PCv13 use and little residual VST circulation. If the authors want to discuss the potential value of 1+1, I would advise also adding potential limitations in this environment and need for high uptake of the booster dose to be implemented (as recommended by the UK).

**Authors’ response:** Thank you for raising this important point. We have added some sentences to the discussion about the relevance of the booster dose in this context.

“Other studies have demonstrated lower than expected direct and indirect effects following PCV13 introduction in Australia, which used a 3+0 schedule, compared to countries using a booster dose, such as the UK (2+1 schedule) and US (3+1 schedule) (Blyth, CID, 2020; Jayasinghe, CID, 2017). As a result, Australia implemented a switch from the 3+0 to 2+1 schedule in 2018. Further research beyond the timeframes available in this analysis is needed to document the longer-term impacts of PCV13 introduction and the schedule change on indirect effects in Australia.”

The supplemental data was very valuable but did not provide specific information to address my questions above.

Overall, this is a very valuable analysis. The largest concern is how the age distributions of the immunized and unimmunized groups compare and whether some adjustment needs to be included as incidence is age sensitive in the first 5 years of life.

**Authors’ response:** Thank you for your positive overall summary. Your concern regarding adjustment for age distribution has been addressed above.

We hope that you will consider our paper for publication in your Journal.

Yours sincerely,

Dr Jocelyn Chan

MBBS BMedSci MPhil(Applied Epidemiology)

Research Clinician, Asia Pacific Health

Murdoch Children’s Research Hospital

Professor Fiona Russell

BMBS GradDip(Clin Epi) Dip Paeds MPHTM FRACP PhD

Director of Child & Adolescent Health PhD Program,

Department of Paediatrics, The University of Melbourne

Senior Principal Research Fellow,

Centre for International Child Health,

WHO Collaborating Centre for Child & Neonatal Health Research and Training,

Department of Paediatrics, The University of Melbourne

Group Leader, Asia-Pacific Health,

Murdoch Children’s Research Institute, The Royal Children’s Hospital, Melbourne
