# Supplementary material for: Levels of pneumococcal conjugate vaccine coverage and indirect protection against invasive pneumococcal disease and pneumonia hospitalisations in Australia: An observational study
Source: PLoS Med. 2021 Aug 3;18(8):e1003733. doi: 10.1371/journal.pmed.1003733 (PMC8376256; doi:10.1371/journal.pmed.1003733)
Supplement: Attachment — Submitted filename: PLOS Med response_12Jul2021.docx [file pmed.1003733.s005.docx]

Monday, 12 July 2021

Dear Dr Raffaella Bosurgi,

Thank you for your considered review of our article and for indicating its likely acceptance. Please find our point-by-point response to each editorial request below.

Requests from Editors:

1. Title: We suggest revising to: “Levels of pneumococcal conjugate vaccine coverage and indirect protection against invasive pneumococcal disease and pneumonia hospitalisations in Australian children: An observational study”

Authors’ response: Amended as requested.

2. Abstract: Please combine the Methods and Findings sections into one section, “Methods and findings”. Please move the sentence describing the main limitations to the end of this section.

Authors’ response: Amended as requested.

3. Abstract: Methods and Findings: Line 16: Please clarify if “addressed” should be “residential address” or similar.

Authors’ response: We have amended the phrase to read “based on residential address at birth only”.

4. Abstract: Lines 22-24: We suggest revising to: “Fifty-percent coverage of PCV7 among children < five years of age was estimate to prevent up to 72.5% (95% CI 51.6-84.4) of PCV7-type IPD among under-vaccinated children, while 90% coverage was estimate to prevented 95.2% (95% CI 89.4-97.8).” or similar.

Authors’ response: Amended as requested.

5. Abstract: Conclusions: Please expand on this sentence, for example, please clarify “...high vaccine coverage is required” by explaining what high coverage is meant to prevent. If space permits, we would suggest an additional sentence, perhaps discussing the implications of your findings for policy considering vaccination schedule, for example.

Authors’ response: “In this study we observed substantial indirect protection at lower levels of PCV coverage than previously described – challenging assumptions that high levels of PCV coverage (i.e. greater than 90%) are required. Understanding the association between PCV coverage and indirect protection is a priority since the control of vaccine-type pneumococcal disease a prerequisite for reducing the number of PCV doses (from three to two). Reduced dose schedules have the potential to substantially reduce program costs while maintaining vaccine impact.”

6. Author summary: Why was the study done? Please revise the third bullet point to: “In this study, we examined associations between PCV coverage and indirect effects within diverse populations within Australia.’

Authors’ response: Amended as requested.

7. Methods: Page 5: Line 21-22: Please revise to “...defined as isolation of S. pneumoniae by culture or detection of nucleic acid from a normally sterile sample…” or similar, as a word seems to be missing.

Authors’ response: We have revised to “...defined as isolation of S. pneumoniae by culture or detection of nucleic acid from a normally sterile site…”.

8. Methods: Thank you for including the RECORD checklist. Please also add the following statement to the Methods: "This study is reported as per the REporting of studies Conducted using Observational Routinely-collected Data (RECORD) guideline (S1 Checklist)."

Authors’ response: Amended as requested (page 7, line 28).

9. Results: Page 12: LIne 17-19: The term "trend" is used here to refer to a non-statistically significant P value. The term trend should be used only when the test for trend has been conducted. Please revise accordingly.

Authors’ response: We have amended the sentence to read: “Additionally, incidence of PCV13-type IPD among under-vaccinated children decreased as PCV13 coverage increased, however confidence interval crossed the null value (adjusted incidence rate ratio [aIRR] 0.989; 95% CI 0.971-1.007; p=0.241). (Fig 4 and S1 File, Table 10).”

10. Results: For adjusted analyses of PCV coverage and incidence, please also confirm the unadjusted results are presented in the manuscript.

Authors’ response: As requested below, we have moved Tables S10 and S11 from the S1 File to the main manuscript – these tables include unadjusted results.

11. Discussion: Page 16: First paragraph: We suggest tempering the language in the paragraph slightly, for example: “Our novel analyses highlight the relatively low levels of coverage required to generate indirect protection in children against IPD and pneumonia hospitalisations in Australia.” could be revised to reflect that findings from these analyses support that a low level of coverage may be required for indirect protection, as compared to what has been previously thought. Also, “indirect effects” is used frequently here and throughout the manuscript. Please consider revising throughout to use an alternative term, or qualifying as “estimated indirect effects” to minimize implications of direct causal effects.

Authors’ response: We have tempered the language in the first paragraph to read:

“The findings from our novel analyses indicate that relatively low levels of coverage are required to generate indirect protection in children against IPD and pneumonia hospitalisations in Australia. These results challenge previous understandings that high coverage is required for substantial indirect protection.”

We have reviewed the manuscript to refer to our results as estimated where appropriate.

We would prefer to keep the term ‘indirect effects’ as this is the accepted terminology to refer to the population-level impacts of vaccines within the vaccine literature (Halloran et al, Am J Epidemiol, 1997). We have clarified this in the introduction and throughout the manuscript, for example:

“Conceptually, the population-level indirect effects of vaccination programs are achieved through sufficient vaccination coverage among age groups primarily responsible for transmission.”

12. Discussion: Page 17: Line 25: Please include the in-text reference citations within square brackets.

Authors’ response: We have added a percentage sign to indicate that the brackets contain a range of vaccination coverage rates rather than citations (i.e., 58-75%).

13. Discussion: Page 16: Line 13: Please place the reference bracket before the sentence punctuation.

Authors’ response: Amended as requested.

14. Discussion: Page 18: Line 32: Please change to “...able to confer…” if that is intended.

Authors’ response: Amended as requested.

15. Page 19: Please remove the “Funding” and “Contributors” sections from the main text, as these will be included with the metadata associated with the manuscript submission.

Authors’ response: Amended as requested.

16. References: Please double check all references for the use of the "Vancouver" style for formatting (in particular, please check Journal title abbreviations, for example, in reference 1, Lancet Global Health should be Lancet Glob Health, and see our website for other reference guidelines: <https://journals.plos.org/plosmedicine/s/submission-guidelines#loc-references>

Authors’ response: Amended as requested.

17. S1 Protocol: In the list of covariates the term “gender” is used, while “sex” is used in the main text (Table 1). The terms gender and sex are not interchangeable (as discussed in http://www.who.int/gender/whatisgender/en/ ); please use the appropriate term.

Authors’ response: We have amended to refer to “sex” throughout both the main text and protocol.

18. Table 1: For the category “sex” please clarify whether percentages are of the total number. For smoking, please provide the responses for “no” if informative.

Authors’ response: We have amended the percentages for “sex” and provided responses for “no” to smoking.

19. Figure 2 and 3: Please provide a descriptive label for the color indicator key used.

Authors’ response: We have added a descriptive label (vaccination coverage) for the colour indicator keys in Figs 2 and 3.

20. Figure 2 and 3 (map images): Please confirm that the appropriate usage rights apply to the use of this map, and please replace with alternative images if necessary. Please see our guidelines for map images: <https://journals.plos.org/plosmedicine/s/figures#loc-maps>

Authors’ response: The following text has already been added below the captions for Figures 2 and 3 in the previously submitted manuscript indicating that appropriate usage rights apply to the use of these maps. Please let us know if you require any additional information.

“Statistical Local Area Digital Boundaries (ASGC 2006) for this map were obtained from the Australian Bureau of Statistics (<https://www.abs.gov.au/>) CC BY 4.0”

21. SI file: Figure 2: In the legend, please include definitions for each abbreviation used in the figure.

Authors’ response: Amended as requested.

22. Supporting information file: Table S10 and S11: Please include these two tables within the Results section (main text) of the paper, if possible, rather than in the supporting information files.

Authors’ response: Amended as requested.

Comments from Reviewers:

Reviewer #2: The authors have satisfactorily responded to each comment in turn.

Authors’ response: Thank you.

Please let us know if we have not adequately responded to any of your requests or if there are any further questions or requests.

Yours sincerely,

Dr Jocelyn Chan

MBBS BMedSci MPhil(Applied Epidemiology)

Research Clinician, Asia Pacific Health

Murdoch Children’s Research Hospital

Professor Fiona Russell

BMBS GradDip(Clin Epi) Dip Paeds MPHTM FRACP PhD

Director of Child & Adolescent Health PhD Program,

Department of Paediatrics, The University of Melbourne

Senior Principal Research Fellow,

Centre for International Child Health,

WHO Collaborating Centre for Child & Neonatal Health Research and Training,

Department of Paediatrics, The University of Melbourne

Group Leader, Asia-Pacific Health,

Murdoch Children’s Research Institute, The Royal Children’s Hospital, Melbourne
